# Impact of Parenting Style on Clinically Significant Behavioral Problems Among Children Aged 4–11 Years Old After Disaster: A Follow-Up Study of the Great East Japan Earthquake

**DOI:** 10.3389/fpsyt.2019.00045

**Published:** 2019-02-19

**Authors:** Takahiro Miki, Takeo Fujiwara, Junko Yagi, Hiroaki Homma, Hirobumi Mashiko, Keizo Nagao, Makiko Okuyama

**Affiliations:** ^1^Department of Global Health Promotion, Tokyo Medical and Dental University, Tokyo, Japan; ^2^Department of Psychosocial Medicine, National Center for Child Health and Development, Tokyo, Japan; ^3^Department of Psychiatry, Iwate Medical University, Iwate, Japan; ^4^Asaka Hospital, Fukushima, Japan; ^5^Fukushima Rehabilitation Center for Children, Fukushima, Japan; ^6^Nagao Mental Clinic, Mie, Japan

**Keywords:** parenting, disaster, corporal punishment, young children, behavior problem, earthquake

## Abstract

**Objective:** The purpose of this study was to investigate the influence of parenting style on children's behavior problems after the Great East Japan Earthquake in 2011.

**Methods:** Participants were children exposed to the 2011 disaster at preschool age (*n* = 163). Data were collected from August 2012 to March 2013, and from July 2014 to March 2015 (2 and 4 years, respectively, after the earthquake), thus participants were aged 4–11 years when assessed. Parenting style was assessed by caregivers using the Alabama Parenting Questionnaire (APQ), which measures parental involvement, positive parenting, poor monitoring/supervision, inconsistent discipline, and corporal punishment in the second year after the disaster. Behavior problems were assessed by caregivers using the Child Behavior Checklist (CBCL), which identifies internalizing, externalizing, and total problems in the second and fourth year after the disaster.

**Results:** The results show that corporal punishment in the second year after the disaster had negative influence on CBCL internalizing score (coefficient: 0.78, 95%CI: 0.12–1.45, *p* = 0.023), externalizing score (coefficient: 0.74, 95%CI: 0.09–1.39, *p* = 0.025), and total score in the fourth year after the disaster (coefficient: 0.85, 95%CI: 0.16–1.55, *p* = 0.016), after adjusted for children's age, sex, the number of trauma experiences, maternal education, the number of siblings, temporally housing experience, and CBCL each scores in the second year after the disaster. Other parenting style did not affect children's behavioral problems.

**Conclusion:** The result suggests that inadequate rearing after a natural disaster had negative impact on the behavior problems of the affected children in 4 years later of the disaster. Specifically, corporal punishment had negative influence on children's behavior problems.

## Key Messages

- The prevalence of children's behavioral problems in the Great East Japan Earthquake affected area is still high.
- The effect of parenting style for children is not clear especially after the disaster.
- This study revealed the association between parental corporal punishment and their children's behavioral problems.
- Support for parents inflicting corporal punishment my reduce children's behavioral problems.

## Introduction

The Great East Japan Earthquake (GEJE) which occurred on 11 March, 2011, had a Richter-scale magnitude of 9.0. The unprecedented natural disaster caused a tsunami of 40.5 m in height (1) and was further complicated by a nuclear accident. As of March 2015, 4 years after the earthquake, the Fire and Disaster Management Agency reported 19,225 deaths, 2,614 missing, and 6,219 injured as a result of the disaster (2). Further, it has an adverse impact on children's mental health (3–7). Usami et al. reported that 42.6% of affected kindergartners to 9th grade showed post-traumatic symptoms (5), and Fujiwara et al. found that one in four children still had behavioral problems 2 years after the disaster (7). Typically, children living in affected areas receive trauma care by a school counselor, or in more severe cases, by child psychiatrist, but not all children received the intervention due to limited resources (8).

Following major traumatic events such as natural disasters, remedies for children's mental disorders and behavioral problem include counseling at school (9) and referral to child psychiatrists. However, such resources are limited by cost, especially after a disaster.

To that end, parenting may be a possible resource as there are no additional costs incurred with the advantage of physical proximity to the children. Nonetheless, there are potential risks to consider, and one of them is corporal punishment which is not prohibited in Japan. Such disciplinary actions may increase post-disaster, and induce further deterioration of behavioral problems among children. Therefore, it is worth investigating the effect of parenting style on child behavior problems after disaster.

McDermott and Cobham (10) reported that children from dysfunctional families showed higher behavior problems 3 months after Cyclone Larry in Australia (10), and Bokszczanin et al. reported that parental social support, family conflicts and parental overprotection are related to post-traumatic stress disorder (PTSD) symptoms 28 months after the Oder Flood in Poland (11). Similarly, perceived parenting styles such as acceptance and firm control moderated the relationship between pre- and post- Hurricane Katrina anxiety symptoms among children aged 6–17 years old (12). Furthermore, a recent review reported that poor parenting coping is associated with adverse mental health among children (13).

To the best of our knowledge, few studies have prospectively investigated the association between parenting style and children's behavior problems after natural disasters among young children. Notably, a reverse causation, that is, behavior problems affecting parenting style, has been suggested (14).

In this study, we investigated the association between parenting style and pre-school children's behavior problems 3 years after GEJE.

## Methods

### Sample

We used data from the GEJE Follow-up for Children study, which recruited caregivers of children who have experienced the GEJE at preschool age (3–6 years old) and their siblings (2–9 years old) in three affected prefectures (Iwate, Miyagi, and Fukushima) (7). We selected municipalities from GEJE-affected areas where one of our team member had personal connections in, and requested for local officials to contact preschools to take part in this study. We recruited caregivers of children from the preschools that have agreed to participate in the study, and conducted the baseline survey.

Baseline data were collected from the recruited children and their caregivers from September 2012 to June 2013, about 2 years after the GEJE. The participants completed questionnaire including parenting practice and other covariates (*N* = 239). Further, follow-up study was conducted from July 2014 to March 2015, about 4 years after the disaster (*N* = 163, follow-up rate: 68.2%). Written informed consent was obtained from the caregivers.

The consent procedures and instruments of this study were approved by the Research Ethics Committee at the National Center for Child Health and Development in Tokyo, Japan.

### Measurements

Parenting practices were assessed by the Alabama Parenting Questionnaire (APQ) (15, 16) in the baseline questionnaire. It consists of 42 full-scale items, each scored by a five-point Likert scale (1 = Never to 5 = Always), and comprises five domains: Involvement (10 items, such as “You play games or do other fun things with your child”), Positive Parenting (6 items, such as “You let your child know when he/she is doing a good job with something”), Poor Monitoring/Supervision (10 items, such as “You do not check that your child comes home at the time she/he was supposed to”), Inconsistent Discipline (6 items, such as “The punishment you give your child depends on your mood”), and Corporal Punishment (3 items, such as “You spank your child with your hand when he/she has done something wrong”). The internal validity of this scale is sufficient in this study (Cronbach's alpha = 0.82, and for each subscale, 0.70, 0.71, 0.73, 0.55, and 0.73, respectively). A higher score denotes lower parenting quality. Children's behavioral problems were assessed by Child Behavioral Checklist (CBCL) in baseline and follow-up study (17). We used total scale and two subscales, namely internalizing scale and externalizing scale. The T score of these two subscales and total scale were computed by standardized distribution among Japanese children, and the children with T scores over 63 were defined as the “clinical range” (18).

Children's traumatic experience related to the disaster was assessed by semi-structured interview directly to the children by child psychiatrist or clinical psychologist in the baseline study. Interviewers were blinded to the profiles of the interviewees, that is, interviewers' were not informed of the APQ score and CBCL scores of the interviewees. The questions about traumatic experiences are follows: lost family member, lost close relative, lost friend, lost house, house being destroyed partially, stayed at shelter, lived in temporary housing, evacuated to relative's house, separated from family member, separated from parents, witnessed tsunami, witnessed someone being swept away by tsunami, witnessed a fire, witnessed a dead body. Other demographics, including living in temporally housing, were collected by a self-reported questionnaire filled by children's caregivers.

### Analysis

First, we analyzed the association between APQ total score and each sub-scale score at baseline and CBCL total score, internalizing score, and externalizing score at the follow-up questionnaire using a bivariate linear regression. Second, association between each APQ subscale and CBCL was investigated, adjusted for each APQ subscale (Model 1). Further, other confounders (child age, gender, number of traumatic experiences related to the GEJE, maternal educational status, number of siblings, living in temporary housing or not, consultation history, and the corresponding CBCL T score at baseline) were adjusted (Model 2). Stata SE 15 (College Station, Texas, StataCorp LP) was used for analysis.

## Results

Demographic characteristics, traumatic experience related to the disaster of children and their caregivers in the affected area, parenting style of caregivers assessed by APQ and baseline behavioral problems assessed by CBCL are shown at Table 1. The mean age of children was 6.8 years old (95% confidence interval [CI]: 6.6–7.0), approximately half of the children were boys (51.5%), and roughly 21.5% of children were the only child. Caregiver's mean age was 36.5 years old (95% CI: 35.7–37.7) and 96.3% of respondents were female. Among them, 5.5% were junior high school graduates, 43.5% were high school graduates, and the rest were some college or higher. Children experienced 2.7 types of traumatic experience (95% CI: 2.3–2.9) related to the disaster on average, and about one-seventh of the participated families lived in temporary house at the time of the follow-up study. The percentage of children who exceeded CBCL cut-off score at baseline, that is, who have clinically significant behavioral problems, was ~20%.

**Table 1:** Characteristics of sample (*N* = 163, baseline data were collected in 2012 and follow-up data were collected in 2014).

**Characteristic**		**Mean or *N***	**SD or %**
Children's age at baseline		6.6	1.4
Children's age at follow-up		8.6	1.4
Children's sex	Male	84	51.5
	Female	79	48.5
Number of siblings	0	35	21.5
	1	75	46.0
	2+	49	30.1
	Missing	4	2.5
Caregiver's age at baseline		36.5	5.9
Caregiver's sex	Male	6	3.7
	Female	157	96.3
Caregiver's educational status	Junior high school graduate	9	5.5
	High school graduate	71	43.5
	Some college	62	38.0
	College+	17	10.4
	Missing	4	2.5
Number of trauma experience related the disaster^*^^a^		2.7	2.0
Living in temporary housing at follow-up	Yes	21	12.9
	No	139	85.2
	Missing	3	1.8
Consultation history about child mental or behavioral problems		29	17.8
Alabama Parenting Questionnaire (APQ) at baseline	Total (range 42–210)	63.4	11.6
	Involvement (range 10–50)	19.3	5.4
	Positive parenting (range 6–30)	10.8	3.1
	Poor monitoring (range 10–50)	13.8	3.9
	Inconsistent discipline (range 6–30)	14.0	3.6
	Corporal punishment (range 3–15)	5.6	1.9
Baseline CBCL clinical range	Total	32	19.6
	Internalizing	35	21.5
	Externalizing	32	19.6
Follow-up CBCL clinical range	Total	12	7.4
	Internalizing	8	4.9
	Externalizing	13	8.0

a *Number of trauma experience related the disaster included lost family member, lost close relative, lost friend, lost house or house being destroyed partially, stayed at shelter, lived in temporary housing, evacuated to relative's house, separated from family member, separated from parents, witnessed tsunami, witnessed someone being swept away by tsunami, witnessed a fire, witnessed a dead body*.

Association between parenting style in baseline (APQ scales) and children's behavioral problems (CBCL scores) in the follow-up survey is shown in Table 2. In crude model, APQ total score was associated with CBCL total behavioral problem T score (coefficient = 0.29, *p* < 0.001), so was APQ parental involvement score (coefficient = 0.47, *p* = 0.002), APQ inconsistent discipline score (coefficient = 0.73, *p* = 0.002), and APQ corporal punishment score (coefficient = 2.25, *p* < 0.001). In Model 1, adjusted for other APQ subscales, APQ parental involvement score and APQ corporal punishment score remain significantly associated with CBCL total behavior problem T score (coefficient = 0.33, *p* < 0.001, and coefficient = 1.93, *p* < 0.001, respectively). The association between APQ inconsistent discipline score and CBCL total behavioral problem T score was no longer significant, suggesting that crude association was confounded by corporal punishment subscale. In Model 2, which was adjusted for covariates, including child age, gender, number of traumatic experiences related to the GEJE, maternal educational status, number of siblings, living in temporary housing or not, consultation history about child mental or behavioral problems, and the corresponding CBCL clinical range at baseline, APQ total score remained significantly associated with CBCL total score (coefficient = 0.21, *p* = 0.01). Further, APQ corporal punishment score was also significantly associated with CBCL total score (coefficient = 1.01, *p* = 0.04) after adjustment of covariates and other APQ subscales.

**Table 2:** Coefficient of CBCL total scale T score in 2016 predicted by APQ scores in 2014.

	**Crude**			**Model 1**			**Model 2**		
	**Coefficient**	**SE**	***p*-value**	**Coefficient**	**SE**	***p*-value**	**Coefficient**	**SE**	***p*-value**
**CBCL TOTAL SCORE**									
APQ total score	**0.29**	**0.69**	**<0.001**	–	–	–	**0.21**	**0.06**	**0.001**
Parental involvement	**0.47**	**0.15**	**0.002**	**0.33**	**0.17**	**0.049**	0.11	0.16	0.49
Positive parenting	0.14	0.27	0.59	−0.34	0.28	0.23	−0.12	0.26	0.65
Poor monitoring/supervision	0.42	0.21	0.05	−0.06	0.23	0.78	0.11	0.22	0.60
Inconsistent discipline	**0.73**	**0.23**	**0.002**	0.25	0.25	0.31	0.4	0.23	0.09
Corporal punishment	**2.25**	**0.40**	**<0.001**	**1.93**	**0.47**	**<0.001**	**1.01**	**0.43**	**0.02**
**CBCL INTERNALIZING SCORE**									
APQ total score	**0.19**	**0.06**	**0.002**	**–**	**–**	**–**	**0.14**	**0.05**	**0.01**
Parental involvement	**0.31**	**0.13**	**0.02**	0.21	0.14	0.16	0.13	0.14	0.35
Positive parenting	0.06	0.23	0.79	−0.23	0.24	0.35	−0.16	0.24	0.49
Poor monitoring/supervision	0.17	0.18	0.95	−0.21	0.20	0.27	−0.10	0.19	0.60
Inconsistent discipline	**0.56**	**0.19**	**0.005**	0.29	0.22	0.19	0.35	0.21	0.09
Corporal punishment	**1.57**	**0.35**	**<0.001**	**1.41**	**0.41**	**0.001**	**0.82**	**0.39**	**0.04**
**CBCL EXTERNALIZING SCORE**									
APQ total score	**0.27**	**0.06**	**<0.001**	**–**	**–**	**–**	**0.22**	**0.06**	**<0.001**
Parental involvement	**0.48**	**0.14**	**0.001**	**0.38**	**0.15**	**0.01**	0.15	0.15	0.33
Positive parenting	0.08	0.25	0.75	−0.4	0.25	0.12	−0.19	0.26	0.45
Poor monitoring/supervision	0.38	0.19	0.05	−0.05	0.20	0.79	0.22	0.21	0.31
Inconsistent discipline	**0.71**	**0.21**	**0.001**	0.30	0.23	0.20	0.36	0.23	0.11
Corporal punishment	**1.95**	**0.37**	**<0.001**	**1.56**	**0.43**	**<0.001**	**0.92**	**0.42**	**0.03**

*Bold correspond to p < 0.05*.

*APQ, denotes Alabama Parenting Questionnaire; SE, denotes standard error*.

*Model 1 adjusted for other APQ subscales*.

*Model 2 adjusted for child age, gender, number of traumatic experiences related to the GEJE, maternal educational status, number of siblings, living in temporary housing or not, consultation history about child mental or behavioral problems, and the corresponding CBCL clinical range at baseline, and other APQ subscales aside from APQ total score*.

As for CBCL internalizing and externalizing score in model 2, similar association was found, that is, APQ total score was significantly associated with CBCL internalizing and externalizing score after adjustment of covariates, and APQ corporal punishment score was also significantly associated with CBCL internalizing and externalizing score after adjustment of covariates and other APQ subscales.

## Discussion

This study revealed two important findings: (1) overall caregiver's parenting style affects behavioral problems among children after the earthquake, and (2) among several parenting styles, corporal punishment was specifically associated with later children's behavioral problem.

To the best of our knowledge, this study is the first to reveal the association between parenting style after a severe natural disaster and children's behavioral problems using prospective cohort design. Previous studies have reported that familial dysfunction, including non-affective responsiveness and non-affective involvement, induces poor mental health among school-aged children (10, 11). However, the same association was not reported among pre-school age children. Moreover, few studies have investigated the impact of parenting on child behavior problem after a disaster (10). In this study, we found an association between parenting style and behavioral problem of offspring after a disaster. This finding would be beneficial for the development of health policy for protection of child mental health. Additionally, corporal punishment is the most significant factor in terms of deterioration of children's behavioral problems. Several studies have revealed that corporal punishment resulted in worse children's behavior (19), especially externalizing problems in other countries (20–22). Similarly, we found a relation between corporal punishment and children's behavioral problem in the stricken area. Children in such areas are known to show more behavioral problems because their resilience might decline after a major disaster (7). In regard to caregivers, their parenting skills might decline after the earthquake. At the same time, the children's behavioral problems would have contributed to the worsening of the caregiver's temper, which in turn led to low-quality parenting and children's behavioral problems. Hence, we postulate that a vicious circle exists between these caregivers and their children.

Further, the protective effect of positive parenting for later total, internalizing, and externalizing behavioral problems among children was implied after adjusting other APQ subscales. However, the protective effect disappeared after adjusting other APQ subscales. The lack of association might be due to the questions assessing positive involvement, for example, “You reward or give something extra to your child for obeying you or behaving well,” or “You tell your child that you like it when he/she helps out around the house,” in which positive parenting was inquired as response to the good behavior of the children. The questionnaire did not ask caregivers whether they involved their children positively even if they had behaved badly.

The findings of this study show some important policy implications. We found that parenting style after disaster, especially corporal punishment, can be a risk factor for behavioral problem, in the ensuing 2 years, regardless of behavioral problems at baseline. Notably, caregivers are the most familiar, accessible, and low-cost potential “care supplier” for all children. Further study is needed to investigate the effect of providing necessary support for caregivers after disaster in reducing behavioral problems among children.

Several limitations need to be mentioned. First, participants of this study may not be representative of the people in the affected area because we recruited participants through voluntary municipalities and preschools (i.e., not random). Further, the participants who dropped out might have more severe conditions than those who remained in the study. Second, we could not assess parenting style before the disaster. Third, both APQ and CBCL were assessed only by the caregivers, so common method bias might exist. Further research using assessment of behavioral problem by others, such as teacher's rating, is needed. Fourth, exposure of traumatic experience due to natural disaster was obtained from interview, which may not be objective. Further research is needed to link the official record of housing damage to validate the self-reported housing damage. Fifth, other forms of child maltreatment, including emotional abuse or neglect, were not included in this study. Therefore, further study is needed to investigate parenting style, including a wider range of child maltreatment on behavioral problems in children.

In conclusion, caregiver's corporal punishment after GEJE was associated with 4 years later behavioral problems among children. After a major disaster, caregivers need to be supported so that they would not use corporal punishment which will cause further deterioration of behavioral problem among children.

## Author Contributions

MO, KN, HH, HM, JY, and TF designed and conducted the GEJE-FC study. TF conceived the current study and finalized the manuscript. TM analyzed the data and wrote the first draft. All authors approved final version of manuscript.

### Conflict of Interest Statement

The authors declare that the research was conducted in the absence of any commercial or financial relationships that could be construed as a potential conflict of interest.
